# Something in the way people move: the benefit of facial movements in face identification

**DOI:** 10.3389/fpsyg.2015.01211

**Published:** 2015-08-11

**Authors:** Andrea Albonico, Manuela Malaspina, Roberta Daini

**Affiliations:** ^1^Department of Psychology, Università degli studi di Milano-BicoccaMilan, Italy; ^2^NeuroMI – Milan Center for NeuroscienceMilano, Italy

**Keywords:** face perception, famous face identification, emotional expressions, typical facial expressions

## Abstract

While the dissociation between invariant aspects of a face and emotional expressions has been studied extensively, the role of non-emotional changeable aspects in face recognition has been considered in the literature rarely. The purpose of the present study was to understand whether information on changeable aspects (with and without emotional content) can help those individuals with poor face recognition abilities (when based on invariant features) in recognizing famous faces. From a population of 80 university students we selected two groups of participants, one with poor performance (experimental group, EG) and the other with good performance (control group, CG). By means of a preliminary experiment, we selected videos of 16 Italian celebrities that were presented in three different conditions: motionless, with non-emotional expressions, and with emotional expressions. While the CG did not differ in the three conditions, the EG showed a significantly better performance in the two conditions with facial movements, which did not differ between each other. These results suggest a role of changeable aspects in the identification of famous faces, rising only in the case invariant features are not analyzed properly.

## Introduction

All fields of cognitive neuroscience have shown evidence that faces are *special* visual stimuli (e.g., Farah et al., 1998) and that we have specific regions for processing them (Michel et al., 1989; Sergent et al., 1992; Kanwisher et al., 1997; Allison et al., 1999). Whether this is *nature* or *nurture* is still a matter of debate, but recent studies have shown that a genetic contribution could occur in both recognition abilities of healthy people and face recognition difficulties of poor recognizers, on the base of participants’ relatives performances (e.g., Schmalzl et al., 2008; Wilmer et al., 2010). For instance, Wilmer et al. (2010) have obtained a correlation of 0.7 between monozygotic twins when performing face recognition tasks, more than two times higher than dizygotic twins (0.29), suggesting that such a specialization is mainly genetically determined.

The numerous demonstrations of specificity in the process of face recognition have led to the development of cognitive models (Bruce and Young, 1986; Haxby et al., 2000; Gobbini and Haxby, 2007), that largely agree about the existence of a dissociation between the perception and processing of the invariant features, that permits the recognition of the identity of a face, and the changeable aspects of a face, which are a major source of information about social context. The anatomo-functional model of Haxby et al. (2000), for example, includes a module that processes the changeable aspects of a face that are connected to all the characteristics important for social interaction, such as analysing eye gaze direction in relation to the orientation of attention, the lip movements (pre-lexical speech perception) associated with oral communication and the facial expressions used to adjust the emotional tone.

In line with Haxby et al. (2000), facial expressions and the identity of a face are processed by two separate routes (involving the superior temporal sulcus, STS, and the fusiform face are, FFA, respectively) after an initial common encoding process and, therefore, are independent of each other. However, some studies have shown that judgments of expression can be modulated by the identity and familiarity of a face (Schweinberger and Soukup, 1998) and vice versa (Sansone and Tiberghien, 1994; Baudouin et al., 2000; Lander and Metcalfe, 2007), suggesting an interaction between the processing of emotion and identity in some circumstances (Gobbini and Haxby, 2007).

This dissociation has been studied mainly by using static faces, with or without emotional expressions. Our everyday experience of faces, however, involves mostly moving faces and the way people move their face can be very “typical.” There are, indeed, facial expressions which are non-emotional, and that may be prototypical of a particular individual, as everyone has some characteristic facial expressions usually. Contrary to emotions, which are often the same and universally recognizable (Ekman and Friesen, 1976; Ekman et al., 1987; Izard, 1994; Elfenbein and Ambady, 2002; but see also Matsumoto, 1992, and Russell, 1994, for the influence of the culture and language on recognition of emotion from facial expressions), these expressions (dynamic facial signature) are idiosyncratic and often without a clear underlying emotional tone (O’Toole et al., 2002).

O’Toole et al. (2002) proposed a model of face recognition in which the processing of the changeable aspects of a face is twofold: the changeable aspects are processed, like many other stimuli of motion, from the dorsal stream (i.e., the pathway in the human visual system that processes spatial and motion information, Ungerleider and Haxby, 1994) and convey information about social content; moreover a structure from motion is obtained which allows identification of dynamic facial signatures and recognition of familiar face.

According to O’Toole et al. (2002), we can learn the idiosyncratic movements of a specific face and we can use them to improve recognition: in fact it has been shown that it is possible to learn to discriminate “artificial” individuals based solely on facial motion information (Hill and Johnston, 2001; Knappmeyer et al., 2001), and faces can be recognized more easily in the presence of facial movements in non-optimal visual conditions (Knight and Johnston, 1997; Lander et al., 1999, 2001; Lander and Bruce, 2000). These two sets of data reflect the two ways in which motion can aid face recognition: the idiosyncratic movements of a specific face could be a different and independent cue to recognition, that is represented regardless of the structural information of the face (supplemental information hypothesis, SIH); on the other hand facial motion could facilitate the perception of the structural model of the face, resulting in an improved recognition (representation enhancement hypothesis, REH). These two theoretical explanations of the motion advantage in face recognition are not mutually exclusive, and various factors, like face familiarity and task demand, may influence the relative importance of the two types of cue (O’Toole et al., 2002).

Several studies have demonstrated the motion advantage (with both rigid and non-rigid facial movements) in different types of tasks involving familiar and unfamiliar face recognition in normal individuals (e.g., Lander et al., 1999; Knappmeyer et al., 2003; Butcher et al., 2011) and in congenital prosopagnosics (Steede et al., 2007; Longmore and Tree, 2013; Daini et al., 2014), but only a small number of studies have considered a further distinction among the latter: facial expressions in fact can include both expressions with an emotional content and expressions without any affective content. Since both these kinds of expressions convey dynamic facial information, it is possible that they are processed in a similar way. However, a recent study by Gobbini and Haxby (2006) has shown that other areas outside the core system for face recognition (i.e., FFA) could be involved in the processing of dynamic expressions, and the involvement of these extra areas could account for the difference usually found between emotional and non-emotional facial expressions, as much as between changeable and invariant features.

It is still an open question whether non-emotional facial expressions can contribute to recognize a face.

The first evidences suggesting that the emotional expressions are processed specifically and independently from the non-emotional expressions in our neural system, came from two studies that showed how these two categories of expressions (emotional and non-emotional) can have different effects on the recognition of static images of unfamiliar faces both in healthy individuals (Comparetti et al., 2011) and in congenital prosopagnosics (Daini et al., 2014). However, to our knowledge, if the difference between emotional and non-emotional expressions could be observed also in recognizing familiar (i.e., famous) faces has not been investigated yet.

In order to explore the role of emotional and non-emotional expressions in face recognition, we selected two groups of participants [a poor recognition or experimental group (EG) and a good recognition or control group, (CG)], different in terms of their ability to recognize faces, and asked them to identify famous celebrities from three different visual presentation conditions: one static and two dynamic (emotional and non-emotional). Moreover, we decided to use videos instead of static pictures because of the dynamic component of facial movement, which is more evident and realistic in this kind of stimuli, compared to static images.

We sought to investigate whether people with poor skills in face recognition can benefit from the information provided by the changeable and dynamic components of a face in order to identify the others. Moreover, we wondered whether any eventual benefit would be strictly related to emotional expressions, or whether it would be due to facial movements and independent of the emotional content, confirming that our visual system analyses and stores non-emotional expressions independently from invariant features and from the content of emotional expressions.

## Materials and Methods

### Preliminary Experiment for Stimuli Selection

The stimuli consisted of 3-s videos of 22 Italian celebrities, selected from television, sports, politics and science, and balanced by gender (11 males and 11 females). We created these three sets of stimuli, in accordance with three different conditions: one in which the face had a neutral expression (Set 1, no expression), a second in which the face assumed an expression of joy (Set 2, emotional expression) and a third in which the face assumed an expression without any affective connotation (Set 3, non-emotional expression). The 3 s videos were taken from longer videos available on the web, by using “VirtualDub 1.9.8.” These cut frames were standardized in size and brightness with a photo editing program (Adobe Photoshop CS4) and reassembled then in a new video (size: 640 × 480 pixels, 22.1° × 34.7°), using the program “Windows Movie Maker.” At the end of the video collection, each celebrity was shown then in three videos, one for each experimental condition and each set (Set 1, no expression; Set 2, emotional expression; Set 3, non-emotional expression) was finally composed by 22 videos.

Prior to the start of the experiment, a separate group of 30 participants, all students at the University of Milan-Bicocca (15 males and 15 females, age = 22.97, SD = 2.19), rated these videos for the main experiment. Each of these participants provided informed consent, in accordance with ethical guidelines by the University of Milan-Bicocca ethical committee. All of them had normal or corrected-to-normal vision and no evidence of neurological or neurophysiological alterations.

The 66 videos were subjected to a rating to confirm the absence of expression in the Set 1 stimuli, the proper recognition of the emotion represented in the Set 2 stimuli and the absence of an affective connotation for the stimuli in Set 3.

Twenty-two videos, selected randomly from the larger sample of 66 videos, were shown to each independent and naïve judge, by using E-Prime 2.0 on the screen of a PC (1280 × 768 pixels, 40.5 × 30.5 cm 60 Hz refresh rate). No time limit was given.

At the end of each video participants of this first experiment were asked to evaluate the presence of a dynamic expression, by using a five level semantic differential scale ranging from 1 to 5, where 1 was the absence of expression and 5 the presence of a very dynamic expression. After, they were also asked to rate how they would classify the previously seen expression of the video (where possible) in terms of emotional content, replying to a multiple-choice question (possible answers: joy, fear, sadness, disgust, anger, surprise, no emotion, other emotion). As a result of this procedure, each video obtained 10 independent ratings. For each video two scores were then calculated: the average score about the dynamic content of the face and the mode of the expression type conveyed by the face. In order to select the videos, different parameter were used: (1) for neutral expression videos, a low intensity (<1.8) and no emotional content; (2) for emotional expression videos: a high intensity (>3.5) and the correct type of expression (joy); and, finally, (3) for non-emotional expression videos, a high intensity (>3.5) and no emotional content. We considered a video satisfactory when at least seven out of 10 judges had given the expected response. The videos that did not meet these requirements were then excluded. Furthermore, if only a video of a celebrity was wrongly evaluated by the judges, all the three videos of that celebrity were then discarded, in order to keep the relationship between the three types of stimuli. Six out of 22 famous people and their videos were thus excluded from the stimuli.

Afterward, the final set of stimuli (see Appendix 1 in Supplementary Material) included videos of the 16 remaining famous people (eight males and eight females) and for each of them, three videos were presented: a neutral expression video, a dynamic emotional expression (joy), and a non-emotional dynamic expression.

### Main Experiment

#### Participants Selection

In selecting participants we sought to identify individuals with poor skills in face recognition, and a CG. For this purpose we recruited (through the University of Milan-Bicocca, Sona System©) from the student population of Psychology at the University of Milan-Bicocca, 63 volunteers who reported no difficulties in face recognition (13 males and 50 females, all right-handed, age range 19–25, mean age 21.59 ± 1.91) and 17 volunteers (1 male and 16 females, all right-handed, age range 19–26, mean age 21.29 ± 2.17) who declared themselves to have difficulties in recognizing familiar faces.

Participation allowed the acquisition of credits and each participant was asked to sign an informed consent for the processing of personal data, in accordance with ethical guidelines by the University of University of Milan-Bicocca ethical committee.

All participants had normal or corrected to normal vision and no evidence of neurological or neurophysiological alterations.

To evaluate the face processing abilities each participant underwent the upright and inverted versions of the Cambridge Face Memory Test (CFMT, Duchaine and Nakayama, 2006; Bowles et al., 2009). This test is composed by three different stages of increasing difficulty, for a total of 72 trials. The inverted version was used to calculate the inversion effect index, IE (Yin, 1969). The average scores of participants with no face recognition difficulties were used to calculate the *z*-scores for each participant; we calculated also the *z*-scores with published control scores (Duchaine and Nakayama, 2006) to confirm the exact selection of the EG with poor face recognition abilities.

None of the 63 participants without face recognition impairment showed a pathological score at the CFMT (i.e., performance lower than 2.0 SD below the mean). Twenty-four out of the 63 participants (2 males and 22 females, all right-handed, age range 19–25, mean age 21.54 ± 1.69) composed our final CG, (good recognizers sample), selected on the basis of a performance above the mean (*z*-score > 0) and on the participants agreeing to come back to undergo the second part of the experiment.

At the end, seventeen participants who referred to having noticed difficulties in face recognition were recruited. On the basis of their scores at the CFMT only 14 of them (1 male and 13 females, all right-handed, age range 19–26, mean age 21.57 ± 3.41) were selected as poor recognizers for the second phase of the experiment (EG, i.e., performance lower than 2.0 SD below the mean). Three participants (C.M., G.T. and P.R.), indeed, were excluded because their performance in the testing phase was not clearly pathological (superior than 2.0 sd above the mean) and, consequently, the presence of a face recognition impairment was not certain (**Table 1**).

**Table 1:** Demographic features of the 24 control subjects and of the 14 subjects impaired in recognizing familiar faces and their performance scores (raw data and *z*-scores) to neuropsychological tests of episodic face recognition.

Experimental group	Age (Y.O.)	Sex	CFMT raw score	CFMT *z*	CFMT *z* (Duchaine and Nakayama, 2006)	IE raw score	IE *z*
A.M.	20	F	38	-2,90^∗^	-2,52^∗^	-2	-2,68^∗^
A.Z.	20	F	41	-2,49^∗^	-2,16^∗^	1	-2,27^∗^
C.C.	19	F	42	-2,35^∗^	-2,04^∗^	10	-1,04
C.R.	21	F	36	-3,17^∗^	-2,77^∗^	6	-1,59
C.S.	19	F	37	-3,03^∗^	-2,65^∗^	-8	-3,50^∗^
C.V.	22	F	42	-2,35^∗^	-2,04^∗^	4	-1,86
E.S.	24	F	37	-3,03^∗^	-2,65^∗^	0	-2,40^∗^
F.C.	26	F	38	-2,90^∗^	-2,52^∗^	-4	-2,95^∗^
L.M.	23	F	40	-2,62^∗^	-2,27^∗^	5	-1,72
M.D.A.	21	F	40	-2,62^∗^	-2,27^∗^	6	-1,59
P.C.	19	F	40	-2,62^∗^	-2,27^∗^	8	-1,31
P.G.	23	M	40	-2,62^∗^	-2,28^∗^	-1	-2,54^∗^
P.V.	20	F	38	-2,90^∗^	-2,52^∗^	5	-1,72
S.R.	25	F	39	-2,76^∗^	-2,39^∗^	0	-2,40^∗^

**CPs**	**21.57 ± 3.41**		**39.1 ± 5.21**			**2.1 ± 4.99**	

**Control group**	**Age (Y.O.)**	**Sex**	**CFMT raw score**	**CFMT *z***	**CFMT *z* (Duchaine and Nakayama, 2006)**	**IE raw score**	**IE *z***

A.B.	22	F	64	0.65	0.61	23	0.73
A.F.M.	23	M	65	0.79	0.73	25	1.01
A.D.M.	22	F	60	0.11	0.13	17	-0.08
A.G.	21	F	69	1.34	1.22	29	1.55
B.S.	20	F	62	0.38	0.37	27	1.28
E.G.	20	F	71	1.61	1.46	34	2.23
F.L.	20	F	64	0.65	0.61	12	-0.77
G.D.S.	22	F	68	1.20	1.10	18	0.05
G.M.	20	F	69	1.34	1.22	14	-0.49
G.R.	23	F	65	0.79	0.73	18	0.05
I.M.	20	F	61	0.24	0.25	24	0.87
L.G.	23	F	63	0.52	0.49	27	1.28
M.B.	23	F	64	0.65	0.61	29	1.55
M.M.	19	M	65	0.79	0.73	19	0.19
M.C.	19	F	63	0.52	0.49	27	1.28
M.C.	22	F	64	0.65	0.61	21	0.46
M.S.	20	F	71	1.61	1.46	13	-0.63
N.P.	25	F	62	0.38	0.37	14	-0.49
S.G.	23	F	61	0.24	0.25	19	0.19
S.B.	21	F	63	0.52	0.49	20	0.32
S.V.	22	F	60	0.11	0.13	23	0.73
S.B.	25	F	69	1.34	1.22	27	1.28
S.T.	20	F	64	0.65	0.61	19	0.19
V.L.	22	F	60	0.11	0.13	18	0.05

**Controls**	**21.54 ± 1.69**		**64.5 ± 3.39**			**21.5 ± 5.77**	

*^∗^Scores falling 2 SD below the mean*.

#### Control Group and Experimental Group

Thirty-eight participants (14 with difficulties in face recognition and 24 controls), selected as described above, participated in this experiment addressing the role of the dynamic aspects of a face in famous people identification.

The group of 14 participants with difficulties in face recognition (1 male and 13 females, mean age of 21.57 years, SD = 3.41, mean education of 16.3 years, SD = 1.9) did not differ from the CG (2 males and 22 females, mean age of 21.54 years, SD = 1.69, mean education of 16.1 years, SD = 1.51) in terms of age (*t*_36_ = –0.045, *p* = 0.963) or years of education (*t*_21_ = –0.284, *p* = 0.777). All participants were right-handed.

#### Stimuli and Procedure

The 48 videos showing 16 Italian famous people (eight males and eight females) in three different conditions (neutral expression, dynamic emotional expression – joy-, and non-emotional dynamic expression) were used for the actual experiment.

The experiment, assembled and driven by E-Prime 2.0, was divided into three blocks, corresponding to the three experimental conditions: neutral (including all the stimuli from Set 1), emotional expressions (with Set 2 stimuli), and non-emotional expressions (with the stimuli from Set 3). The presentation of the stimuli within each set was randomized and the order of presentation of the three blocks was counterbalanced among the participants (three different sequences). The participants were sited at a distance of 40 cm from the PC screen and the videos were shown in a central position on the screen, after 500 ms of a black mask. Participants were asked to watch the video, and after to provide the name of the famous person or, alternatively, to give any biographical information that was linked to that face. No time limit for the response was given. Thus, for each condition the maximum score was 16, corresponding to the correct identification of all celebrities. At the end of the experimental session, participants were asked whether they knew all the celebrities used in the experiment, by providing them the names of the celebrities. None of the celebrities was unknown to the participants.

## Results

The number of correct famous face identifications in the three conditions were submitted as dependent variable to an analysis of variance with two between-subjects main factors “Group” (two levels: EG and CG) and “Sequence” (three levels: neutral–emotional–non-emotional (N–E–NE), non-emotional–neutral–emotional (NE–N–E) and emotional–non-emotional–neutral (E–NE–N) and one within-subjects main factor: “Stimulus Condition” [three levels: neutral (N), emotional (E) and non-emotional (NE)].

The analysis of variance showed a significant main effect of “Group” [*F*(1,32) = 8.912; *p* = 0.005; η^2^ = 0.28] and a significant main effect of “Stimulus Condition” [*F*(2,64) = 6.474; *p* = 0.003; η^2^ = 0.20]. The main effect of “Sequence” [*F*(2,32) = 0.833; *p* = 0.444; η^2^ = 0.052] was not significant. The “Group” by “Stimulus Condition” interaction was significant [*F*(2,64) = 7.387; *p* = 0.001; η^2^ = 0.23], as was “Stimulus Condition” by “Sequence” [*F*(4,64) = 3.375; *p* = 0.014; η^2^ = 0.21]. The “Group” by “Sequence” [*F*(2,32) = 1.751; *p* = 0.190; η^2^ = 0.109] and the “Group” by “Sequence” by “Stimulus Condition” [*F*(4,64) = 0.511; *p* = 0.728; η^2^ = 0.03] interactions were not significant.

The EG, made up of participants who showed difficulties with unfamiliar face recognition tests, had a worse performance than the control participants even in the famous faces identification task (9.35 vs. 11.54 correct answers, respectively). The main effect of Stimulus showed that, overall, the neutral condition (10.14) was significantly more difficult than the non-emotional condition (10.78; *p* = 0.009), which instead did not differ from the emotional condition (10.42).

**FIGURE 1:**
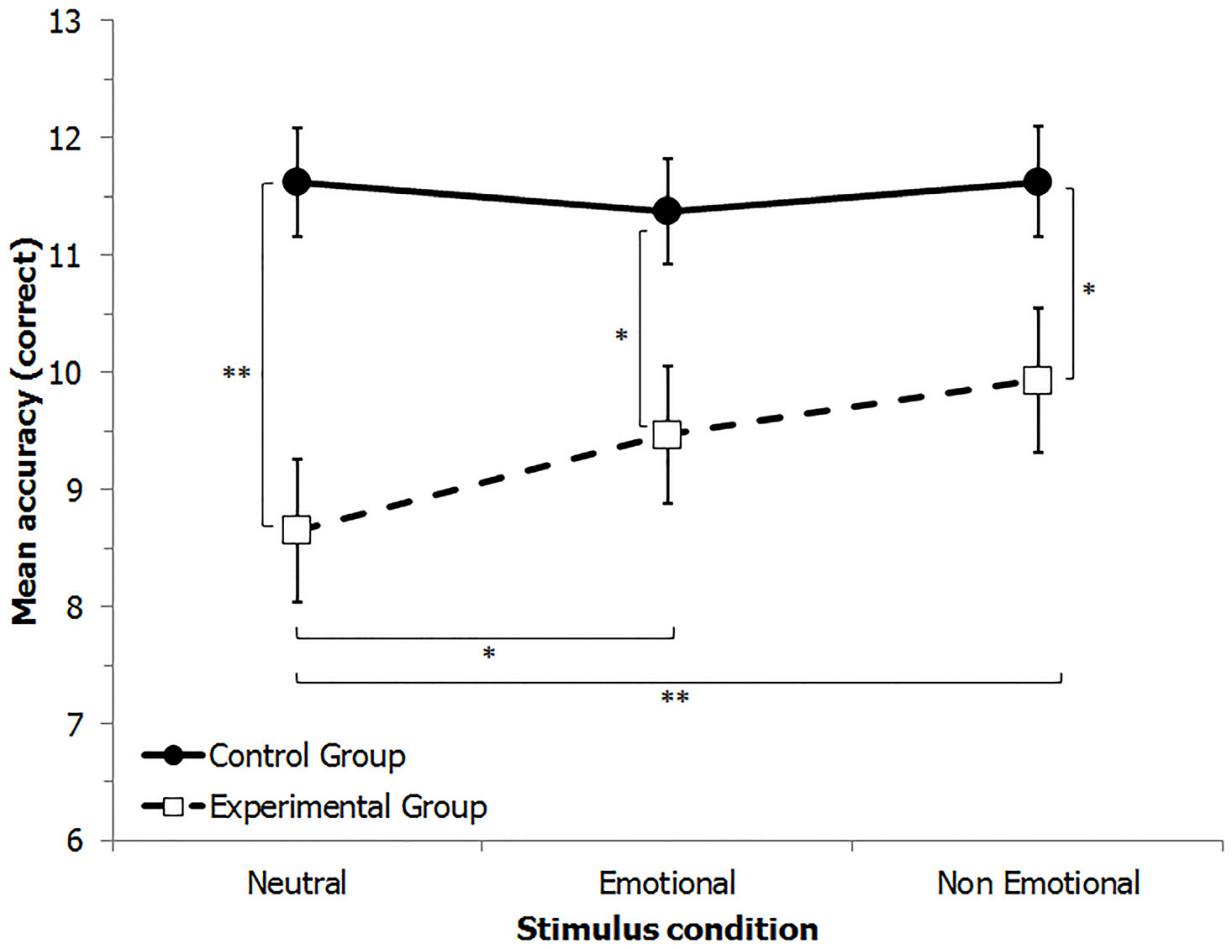
**Mean number of correct identifications of famous people in the three experimental conditions: with neutral faces, with emotional expressions and with non-emotional expressions in the experimental (EG) and the control (CG) groups**. Vertical lines indicate ±1 SE. ^∗^*p* < 0.05; ^∗∗^*p* < 0.01.

The significant “Group” by “Stimulus condition” interaction (**Figure 1**) was further explored by *post hoc* multiple comparisons (Bonferroni). The EG exhibited a significantly lower performance then the CG in all three conditions (neutral: *p* = 0.001; emotional: *p* = 0.015; non-emotional: *p* = 0.036). Moreover, in the EG the neutral condition was significantly lower than the other two conditions (*p* = 0.019 and *p* = 0.001) and the emotional was not significantly different from the non-emotional condition (*p* = 0.222). No differences were present for the CG in the three stimulus conditions (all *p* > 0.05).

These results are in line with the hypothesis that individuals with poor face recognition skills can be helped by the presence of facial movements: in fact their performance improved in the two dynamic conditions, independently of the emotional content. Not only did the presence of an emotion not improve the performance, but even facial movements without emotional content showed a small improvement with respect to the emotional condition, even if not statistically significant. One possible explanation for these findings could be that it is easier to extract the typical expressions of a person without processing the affective meaning of them.

Also the significant “Stimulus condition” by “Sequence” interaction was further explored by *post hoc* multiple comparisons (Bonferroni). In the first sequence N–E–NE, the first presentation (neutral) was significantly lower than the third (non-emotional; *p* = 0.007), while in the third sequence E–NE–N the first presentation (emotional) was significantly lower than the second (non-emotional; *p* = 0.011). In general, the first condition was always lower than the other two, and this difference was not significant only in the second sequence, between the first dynamic condition and the second neutral condition. Nevertheless, the absence of any interaction between the group and the sequence confirms that the effects we were looking for and that we obtained, were independent of the “obvious” effect of the sequence.

## Discussion

We seleceted two groups on the basis of their differences in performing face matching and recognizing judgments during a neuropsychological assessment. These two groups were also significantly different during an experimental task involving the identification of famous faces. The group with poor face recognition skills was impaired in recognizing famous people, even if external cues, such as hair and upper body parts, were available. The difference in performance between the two groups was not only quantitative, but also qualitative. Only the performance of the group with poor face recognition, indeed, showed an effect of stimuli condition. In particular, their performance was significantly worse for neutral faces than for the two dynamic conditions.

The presence of facial expressions did not help in recognizing famous people in the case of the group with good recognition: this was probably due to the fact that they are already at ceiling in recognizing faces from their invariant features, so the expressions were of no help. As shown by other authors (for a review, see O’Toole et al., 2002), facial motion is unlikely to benefit identity recognition in normal conditions, but dynamic information can be helpful in difficult conditions (like poor illumination, noise, etc.) and can improve face recognition (Steede et al., 2007; Longmore and Tree, 2013). We showed that the difficult conditions can relate not only to the external factors but also to the limitations of the participants.

Indeed, only the poor recognition group derived benefits from the celebrities’ facial movements, while the group with good recognition did not derive any benefit from the presence of expressions in famous faces.

A possible explanation is that both groups could use facial movements, but the good recognizers already showed the maximum performance with the neutral condition, because their ability to process facial invariant aspects was preserved and more efficient than facial movements processing. An alternative explanation is that people with difficulties in the processing of invariant aspects of faces learned to use facial movements cues to compensate for their difficulties, becoming better than the controls in using those information. Unfortunately, starting from our data we are not able to disentangle between these two hypotheses and new studies will be necessary for a better understanding of the interaction and integration of the two systems for face recognition.

The order of presentation had an effect on performance, so that the same celebrity presented a second or third time was better recognized than on the first occasion, independently of the group. Moreover, we did not tested all possible sequences and this can be a limitation of the present study. Nonetheless, the fact that the sequence did not interact with the difference observed between groups in the three conditions, suggests that such an effect was independent of the effects that we were interested in.

Finally, the improvement in performance in the poor face recognition group was not limited to the emotional expressions condition, but it was also present in the non-emotional expressions condition. This is consistent with the idea that the performance of individuals with difficulties in face recognition is not improved by the emotional content, but it is improved by the changeable aspects, which were present in both conditions of our experiment. This does not mean that the two aspects of faces, emotional expressions and non-emotional expressions, do not differ from each other, but it suggests that the way people move their face, regardless of whether they are speaking or smiling, is typical, and can be separately processed, stored and recovered. It is possible that when a person moves his face to express some emotional content, his/her expressions are as typical as when the facial movements do not contain any emotional content.

Our results showed that facial expressions can improve face recognition also in the case of famous faces, in addition to the already demonstrated case of unfamiliar faces (Comparetti et al., 2011; Daini et al., 2014). On the other hand, the different results we obtained, compared with previous studies, may be attributed to the use of a different presentation of the stimuli. In fact, while previous studies (Comparetti et al., 2011; Daini et al., 2014) used static images and found that emotional and non-emotional expressions can have different effects during a recognition task, in our study we found that both types of expressions can have the same impact on identification of naturally moving faces of famous people. This result could be due to the fact that videos stimuli convey more dynamic information than static pictures, allowing the participants to focus more on the dynamic information itself and so minimizing the differences between the emotional and non-emotional content. Moreover, we did not ask to detect the expression, but to identify the person, independently from the presence of a facial expression. Nevertheless, one possible limitation of our study is the small sample size of the group with poor face recognition abilities, which, however, is strictly related to the complexity of finding and recruiting those participants.

Our results support the hypothesis that our visual system analyses and stores non-emotional expressions independently from invariant features and emotional content, as suggested only by O’Toole et al.’s (2002) model, and that in poor face recognizers the changeable aspects might be preserved and might help recognition, giving supplemental information for face recognition (independent from the structural information of the face, accordingly to the SIH). Such information (together with the invariant features of the face and all biographical information) is part of what defines the identity of a person, and is the basis of facial imitation.

## Conclusion

The facial movements are not particularly useful for individuals with good performance in face processing when they have to identify famous people in optimal condition because they can already rely on invariable aspects, and this appears to be the main pathway for identification. On the other side, individuals with poor face recognition skills can benefit from facial movements in order to identify well-known celebrities, suggesting that motion information can be extracted from an image sequence or a video and can act as a cue for identification. It appears that poor recognizers could have coded and learned face information by relying to a more preserved processing of changeable aspects, compared to a less efficient processing of invariant aspects, and that they could use motion as a supplemental cue in face recognition. On the other hand, the lack in performance of our EG, despite the advantage of facial dynamic information, suggests the greater relevance of invariant features in face recognition and its deficiency in those participants.

Our results are relevant for both theoretical and practical reasons. They support the hypothesis of a system in our brain that is able to process, learn and recover typical facial expressions, independent of emotional content. Such a system seems to be preserved in individuals who show poor face recognition abilities (and this could be extended to individuals with congenital prosopagnosia), and it is possible that they can be trained, possibly early in youth, so as to improve their ability to interact with others in everyday life.

## Conflict of Interest Statement

The authors declare that the research was conducted in the absence of any commercial or financial relationships that could be construed as a potential conflict of interest.
