# Supplementary material for: Something in the way people move: the benefit of facial movements in face identification
Source: Front Psychol. 2015 Aug 11;6:1211. doi: 10.3389/fpsyg.2015.01211 (PMC4531343; doi:10.3389/fpsyg.2015.01211)
Supplement: Supplementary file 1 [file Data_Sheet_1.DOCX]

**Appendix 1**

Names of 16 famous people selected for the dynamic face experiment

| Arisa |
| --- |
| Belén Rodríguez |
| Ilary Blasi |
| Cesara Bonamici |
| Elisabetta Canalis |
| Anna Falchi |
| Margherita Hack |
| Cristina Parodi |
| Pier Luigi Bersani |
| Paolo Bonolis |
| Massimo D'Alema |
| Giovanni Floris |
| Dario Franceschini |
| Luca Laurenti |
| Ignazio Marino |
| Christian De Sica |
